# Use of Targeted Exome Sequencing for Molecular Diagnosis of Skeletal Disorders

**DOI:** 10.1371/journal.pone.0138314

**Published:** 2015-09-18

**Authors:** Daniel L. Polla, Maria T. O. Cardoso, Mayara C. B. Silva, Isabela C. C. Cardoso, Cristina T. N. Medina, Rosenelle Araujo, Camila C. Fernandes, Alessandra M. M. Reis, Rosangela V. de Andrade, Rinaldo W. Pereira, Robert Pogue

**Affiliations:** 1 Programa de Pós-Graduação em Ciências Genômicas e Biotecnologia, Universidade Católica de Brasília, Brasília, Distrito Federal, Brazil; 2 Núcleo de Genética da Secretaria de Saúde do Distrito Federal, Brasília, Distrito Federal, Brazil; 3 Curso de Medicina, Universidade Católica de Brasília, Taguatinga, Distrito Federal, Brazil; 4 Departamento de Tecnologia, Laboratório Multiusuário Centralizado para Sequenciamento de DNA em Larga Escala e Análise de Expressão Gênica, Faculdade de Ciências Agrárias e Veterinárias, Universidade Estadual Paulista, Campus Jaboticabal, Jaboticabal, São Paulo, Brazil; Emory University School Of Medicine, UNITED STATES

## Abstract

Genetic disorders of the skeleton comprise a large group of more than 450 clinically distinct and genetically heterogeneous diseases associated with mutations in more than 300 genes. Achieving a definitive diagnosis is complicated due to the genetic heterogeneity of these disorders, their individual rarity and their diverse radiographic presentations. We used targeted exome sequencing and designed a 1.4Mb panel for simultaneous testing of more than 4,800 exons in 309 genes involved in skeletal disorders. DNA from 69 individuals from 66 families with a known or suspected clinical diagnosis of a skeletal disorder was analyzed. Of 36 cases with a specific clinical hypothesis with a known genetic basis, mutations were identified for eight cases (22%). Of 20 cases with a suspected skeletal disorder but without a specific diagnosis, four causative mutations were identified. Also included were 11 cases with a specific skeletal disorder but for which there was at the time no known associated gene. For these cases, one mutation was identified in a known skeletal disease genes, and re-evaluation of the clinical phenotype in this case changed the diagnoses from osteodysplasia syndrome to Apert syndrome. These results suggest that the NGS panel provides a fast, accurate and cost-effective molecular diagnostic tool for identifying mutations in a highly genetically heterogeneous set of disorders such as genetic skeletal disorders. The data also stress the importance of a thorough clinical evaluation before DNA sequencing. The strategy should be applicable to other groups of disorders in which the molecular basis is largely known.

## Introduction

Achieving a definitive diagnosis in groups of genetically heterogeneous diseases with overlapping phenotypes is complicated and in some cases may fail to reduce the number of genes to be screened for mutations to a tractable number [1, 2]. Diagnostic tests for single gene diseases can focus on mutation analysis for specific causative mutations, such as for achondroplasia [3] or cystic fibrosis [4], as well as resequencing and analysis of specific genes in which mutations may lead to similar phenotypes [5]. Through the development and dissemination of Next-Generation Sequencing (NGS) technologies, the broader sequencing of the complete or partial human genome has become accessible for use in the diagnosis of genetic diseases [6, 7] achieving a success rate for clinical genome and exome sequencing of around 25% [8–10].

While exome sequencing has become more common, including for diagnostic purposes [11–14], analysis of mutations in heterogeneous disease groups in which the associated genes are largely known has been successfully carried out with a limited number of genes using the targeted panel sequencing approach [2, 15–19]. This approach uses specific gene panels, focusing only on known loci, allowing increased quality, fidelity, performance and speed. With this approach it is also possible to sequence samples in multiplex, further decreasing cost due to increased efficiency [2, 19, 20].

Bone development and growth are regulated by a range of genetic factors. Any genetic defect that affects this regulation may generate a number of clinical conditions affecting skeletogenesis [21, 22]. According to the most recent nosology, skeletal disorders include more than 450 distinct diseases. These diseases have been organized into groups [23], though this classification is not perfect due to overlap with regard to clinical, radiological and genetic characteristics. So far, more than 300 genes have been recognized as causing one or more skeletal disorders [23–25]. Within this group of diseases are many rare phenotypes that few clinicians have encountered and can readily recognize. Difficulties in diagnosis are reflected in the various efforts that have been made to classify the disorders according to clinical, radiological or molecular characteristics [22, 26].

In this context, the present work describes the use of targeted exome sequencing to analyze 309 known skeletal disorder genes in 66 families with a known or suspected diagnosis. Causative mutations were identified in 13 cases, including four cases of suspected skeletal disorder that were definitively diagnosed, and another two cases that had their original diagnoses modified through the molecular data.

## Materials and Methods

### Ethics statement

All procedures used in this study were conducted according to the principles of the Declaration of Helsinki. The Institutional Review Board and the Ethics Committee of Catholic University of Brasília approved the protocols used (CEP/UCB 056/2011). All participating patients and family members signed an informed consent declaration.

### Case selection

The cases used for validation of the panel were archived cases from the Genetics Service of the Health Secretariat of the Federal District, Brazil. Cases were accepted if they had a diagnosis of a specific skeletal disorder, or if a skeletal disorder diagnosis was suspected or less specific (for example, osteodysplasia, chondrodysplasia). The basic requirement was evidence of defect of bone formation or growth based on clinical evidence of short stature, skeletal deformity or abnormalities observed on radiographs, or any diagnosis encountered in the genetic skeletal disorders nosology [23]. Sequencing was performed for a total of 69 samples. Of these, three were parents of affected offspring, giving a total of 66 genetically independent cases (S1 Table). Three of the 69 samples were sequenced twice, for a total of 72 sequenced libraries.

All patients were from the general Brazilian population, a multiethnic and highly admixed population with a heterogeneous genetic profile. Estimates of its ancestry unveiled a major contribution of European ancestry (>60%) followed by African ancestry and a minor (<10%) native Brazilian Amerindian ancestry [27–30].

Among the cases included was a subset of 36 cases (subgroup 1, S1 Table) with a firm initial diagnosis. In addition, 20 cases were classified as having a suspected skeletal disorder (subgroup 2, S1 Table), mainly being diagnosed with ‘osteodysplasia’. The remaining 11 cases (subgroup 3, S1 Table) were several cases with diagnoses of skeletal disorders for which there was no known associated gene, such as Fibular Aplasia, Tibial Campomelia, and Oligosyndactyly (FATCO) syndrome, Marden-Walker syndrome, Goldenhar syndrome and Catel-Manzke syndrome. These were included based on the possibility of the causative mutation occurring in a gene that is on the panel. Furthermore, there were several cases with specific diagnoses for which one or more loci are known, but these loci do not account for 100% of cases. These included a number of cases of craniosynostosis.

### Design of the panel

The principal information source for inclusion of genes on the targeted exome panel was the 2010 skeletal disorder nosology [23]. All genes listed in this publication were included on the panel. In addition, a review of the more recent literature was performed to include skeletal disorder-associated genes identified since the publication of the 2010 nosology [11, 31–70]. In addition to the coding regions of skeletal disorder genes, regions that cause skeletal disorders when deleted, such as the *SOX9* regulatory region associated with campomelic dysplasia were included in the panel.

In total, we selected 309 genes known to be involved in genetic disorders of the skeleton (S2 Table). The sequences corresponding to these genes were uploaded to the Web-based probe design tool DesigStudio (Illumina, Inc.), resulting in generation of a total of 7,004 capture probes over 4,930 targets, with a predicted coverage of 99% of these regions, estimated sequencing success ≥95% and a panel size of approximately 1.44 Mb. The coordinates of the sequence data are based on UCSC hg19. For the probe design, the parameters were set to select all exons of each gene, plus 50 bp of flanking intronic sequences for detection of splicing mutations.

### Sample Preparation

Sample preparation was performed according to the manufacturer’s instructions (Illumina TruSeq DNA Sample Preparation V2 Guide low-throughput Rev A). In brief, the quality of each sample was verified by gel electrophoresis prior to fragmentation. Fragmentation of genomic DNA samples was carried out either by nebulization or ultrasonication.

During sample preparation, bar-coding index tags were ligated to the fragments in order to permit simultaneous sequencing of 12 samples. After the DNA library preparation, the validation of the libraries was performed by checking the size of the fragments of each genomic library by agarose gel and subsequent quantification using the Qubit dsDNA BR Assay Kit (Life Technologies, Waltham, MA, USA).

### Capture and sequencing

Capture of the sequences of interest was performed according to the manufacturer's instructions (Illumina TruSeq Enrichment Guide Ver J, Illumina, San Diego, CA, EUA). Briefly, the DNA libraries were grouped into pools of twelve libraries. Then, a maximum of 500 ng of DNA from each pooled library were diluted (totaling a maximum of 6 μg DNA) and mixed with the capture probes designed during construction of the custom gene panel. Two hybridization steps were carried out to ensure enrichment of the regions of interest, and a final enriching PCR was performed to amplify the fragments. After this, validation of the pooled libraries was performed by quantifying and verifying the size of the enriched fragments.

The Bioanalyzer High Sensitivity chip (Agilent Technologies, Santa Clara, CA, USA) was used to check the size range of the enriched fragment libraries. The quantification of the library was performed by either fluorometry with the Qubit dsDNA HS Assay Kit (Invitrogen, Carlsbad, CA, USA) or quantitative PCR (qPCR) using the KAPA Library Quantification Kit for Illumina sequencing platforms (KAPABIOSYSTEMS, Wilmington, MA, EUA) using the average fragment size of each library according to the Bioanalyzer (464 bp).

### Sequencing

The preparation of the pooled libraries for sequencing on the MiSeq platform was performed according to the manufacturer's instructions. Sequencing was carried out using a 2x150 cycle protocol (total yield of up to 3.5 Gb of sequence), with a maximum theoretical coverage of at least 200X per sample and 120X expected mean coverage.

### Data Analysis

The Ilumina MiSeq reporter software was used to generate *fastq*.*gz* output files. Next, the NextGENe software [16, 18–20, 71–73] (Softgenetics, State College, PA, EUA) was used for alignment, SNP/Indel detection, coverage statistics and detection of variations in two databases, dbSNP (v. 135) and dbNSFP (v. 2.0). The dbNSFP compiles six prediction algorithms (SIFT Polyphen2, LRT, MutationTaster, MutationAssessor and FATHMM) three conservation algorithms (PhyloP, GERP++ and SiPhy; the greater the score, the more conserved the site) and information including allele frequency observed in the data from the 1000 Genomes Project phase 1 and NHLBI Exome Sequencing Project [74, 75]. The alignment was performed using the regions of interest (ROI) of the gene panel (~1.44 Mb) as a reference template.

For focused molecular analysis that aims to assist in diagnosis, false negative results due to missed nucleotides must be avoided. As such we determined the base-by-base coverage in the target regions before excluding a candidate gene. The data were plotted on a scatter plot (S1 Fig).

**Fig 1:**
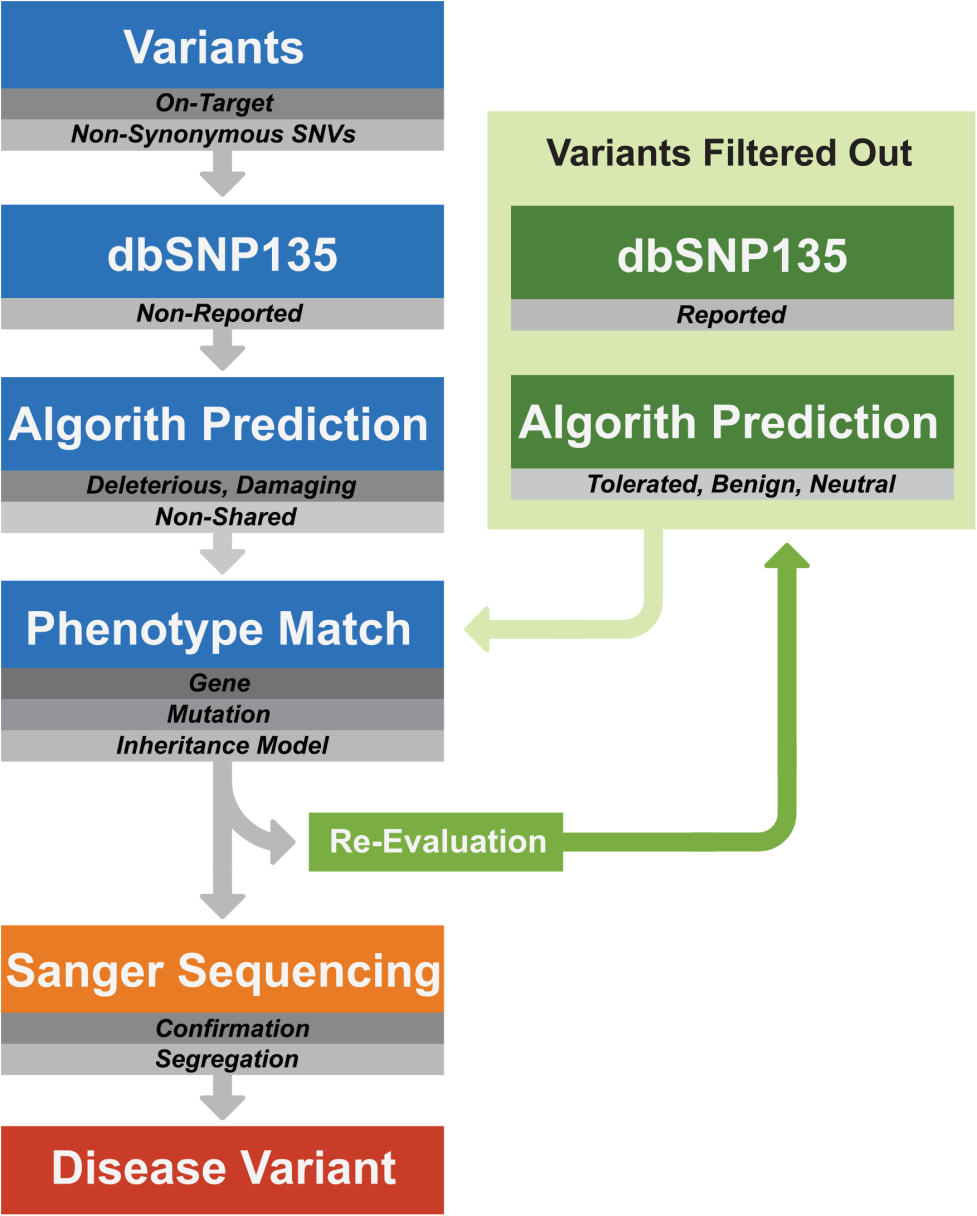
Analysis of the molecular data obtained by targeted exome sequencing.

Variants predicted to be deleterious by at least two algorithms, and affecting conserved amino acids were prioritized. Variants found in each sample were evaluated and correlated with the monogenic nature of the predicted phenotypes of each case. After this, according to the diagnosis in each case, candidate mutations for each sample were selected. A database search on the HGMD (Human Gene Mutation Database) was used to determine whether the variants had been previously reported. If there was no mutation in the expected gene, all filtered variants were matched with HGMD and OMIM data with a view to modifying the original diagnoses (Fig 1).

Finally, the results were discussed with the medical collaborators and molecular diagnosis was defined in cases in which the candidate mutations matched with diagnosis.

### Validation of Mutations by Sanger Sequencing

All candidate mutations found by targeted exome sequencing were confirmed using Sanger sequencing. In addition to the patients’ DNA sample, the samples of their relatives were used for segregation analysis. Also, a panel of population control DNA samples was used. The 1000 Genomes Project, Exome Variant Server, and the ExAC Browser were searched in order to determine whether the variants were previously reported. However, since these databases do not contain cohorts of Brazilian controls, we also performed analysis of allele frequency in a group of 210 chromosomes from 105 normal individuals of the Brazilian population (collected in the Federal District, Brazil) in order to rule out the possibility of the identified variants being common polymorphisms [76]. Since the cases were all unrelated, each case can also serve as a control for the others, thus increasing the total number of control chromosomes for each mutation. All validated causative mutations were deposited in the ClinVar repository (SCV000223901-SCV000223913).

## Results

### Sequencing

Fragmentation was successfully carried out by nebulization or by ultrasonication, with size range of the fragments from each library ranging between 300 and 1000 bp. Each run yielded at least 3.4 Gb of high-quality data (≥ Q30).

The percentage of sequences on target for all samples was greater than 70%, and the average coverage of the samples proved to be satisfactory at 131X (+/- 14.7), higher than the expected coverage. The percentage of regions of interest with coverage greater than 10X was above 90% (93.4 +/- 0.05%) and with coverage greater than 20X was just under 90% (89.3 +/- 0.08%).

Few targets showed coverage below 1X. Of the total of 4,930 targets, 56 (1.13%) were not sequenced in at least 50% of the samples. Of these targets, 47 were located in the first exons or UTR of the genes. This consistency in the data indicates a failure at the capture step of these sequences, probably caused by sequence complexity and GC content.

When analyzed base-by–base, all samples showed a coverage distribution that reflected high coverage across all nucleotides, with a peak in the center of the sequenced region (S1 Fig). It was found that the distribution pattern of the coverage was maintained across all samples that had high average coverage, varying only in intensity range due to the coverage of the specific sample.

For each sample, compared to the reference genome (hg19), an average of 200 (+/- 7) variants were found, including silent (60%), and missense or nonsense (40%) variants. After filtering the silent variants, 90% were alterations found in dbSNP. In the initial filtering, these were removed and only the remaining variants were analyzed. However, since dbSNP has a channel with pathological variants, the data were also searched for candidate mutations in this database (Fig 1). The mutation filtering process also included the removal of variants not found in dbSNP but that were shared among two or more cases from different families and with different phenotypes, presuming that these were previously unreported SNPs [2] present in the Brazilian population.

### Individual samples

At the end of the initial filtering stage, 5–15 possible pathological mutation candidates were identified in each case. The variants were then considered in the context of the clinical phenotype to determine whether a definitive molecular diagnosis could be reached. This analysis led to a definitive molecular diagnosis in 13 cases, with 6 alterations previously reported in HGMD as causative mutations concordant with the phenotype (Table 1). Eight cases (22%) with mutations were from the cohort of 36 cases (subgroup 1, S1 Table) with a firm initial diagnosis. This included one case with an initial diagnosis of Sotos syndrome (R121), but on finding a mutation in the *EZH2* gene the case was re-evaluated and determined to be Weaver syndrome. For the case diagnosed as Hajdu-Cheney syndrome (sample R41), no mutation was found in the expected exon (exon 34) [77]. The patient’s affected mother (sample R41_1) was also sequenced, with the same result. Thus we rule out Hajdu-Cheney syndrome caused by *NOTCH2* exon 34 mutation. The mother and child shared several candidate variants, though none related to the Notch pathway, and none that were obviously related to the phenotype. They are being clinically re-evaluated, however we cannot rule out the possibility of a second Hajdu-Cheney syndrome locus.

**Table 1:** Summary of causative mutations identified after molecular analysis in the investigated cases.

Sample ID	Definitive Medical Diagnosis	Gene ID	NM Number	cDNA Level Change	Protein Level Change	NGS Coverage	HGMD*	MIM Number	Classification^#^
**R6**	Spondylocarpotarsal Synostosis	*FLNB*	NM_001457.3	c.1945C>T	p.(Arg649Ter)	89X	CM040999	272460	Pathogenic
**R34**	Neurofibromatosis-Noonan syndrome	*NF1*	NM_000267.3	c.625C>T	p.(Gln209Ter)	139X	NA	162200	Pathogenic
**R43**	Geleophysic dysplasia 2	*FBN1*	NM_000138	c.5284G>A	p.(Gly1762Ser)	99X	CM115510	614185	Pathogenic
**R47**	Ectrodactyly, Ectodermal Dysplasia, and Cleft Lip/Palate syndrome 3	*TP63*	NM_001114978.1	c.740A>G	p.(His247Arg)	133X	CM100075	604292	Pathogenic
**R82**	Apert syndrome	*FGFR2*	NM_022970.3	c.755C>G	p.(Ser252Trp)	86X	CM970526	101200	Pathogenic
**R85**	Nail-patella syndrome	*LMX1B*	NM_001174146.1	c.306C>G	p.(Tyr102Ter)	35X	NA	161200	Likely Pathogenic
**R99**	Cleidocranial dysplasia	*RUNX2*	NM_001015051.3	c.476G>A	p.(Gly159Asp)	125X	NA	119600	Likely Pathogenic
**R121**	Weaver syndrome	*EZH2*	NM_152998.2	c.149T>C	p.(Leu50Ser)	194X	NA	277590	Likely Pathogenic
**R122**	Townes-Brock syndrome	*SALL1*	NM_002968.2	c.949C>T	p.(Pro317Ser)	28X	NA	107480	Likely Pathogenic
**R150**	Hypochondroplasia	*FGFR3*	NM_001163213.1	c.1626C>G	p.(Asn542Lys)	96X	CM950475	146000	Pathogenic
**R156**	Wolcott-Rallison syndrome	*EIF2AK3*	NM_004836.5	c.1192C>T	p.(Gln398Ter)	35X	CM113379	226980	Pathogenic
**R163**	Spondyloepiphyseal Dysplasia, Congenital type	*COL2A1*	NM_001844.4	c.3301G>A	p.(Gly1101Arg)	294X	NA	183900	Likely Pathogenic
**R174**	Trichrhinophalangeal syndrome I	*TRPS1*	NM_014112.4	c.1693C>T	p.(Gln565Ter)	177X	NA	190350	Likely Pathogenic

*HGMD Access code.

^#^Variant Classification according to the ACMG and AMP guidelines [78]. NA: Not applicable.

From the cohort of 20 cases (subgroup 2, S1 Table) with a suspected skeletal disorder, a molecular diagnosis could be finalized for four (20%) cases. This subgroup also included a trio (samples R169, R169_1, R169_2), being a case of idiopathic shortening of the metatarsals, and unaffected parents and no candidate mutations were found. This may be a case of a new mutation. Finally, one case originally from the group of 11 samples (subgroup 3, S1 Table), that was initially thought to have a phenotype with no known gene at the time, was re-evaluated and determined to be Townes-Brock syndrome. Here, the patient was suspected to have Goldenhar syndrome, but in light of a mutation in the *SALL1* gene, and the re-evaluation of the patient phenotype, the diagnose was changed.

### Confirmation by Sanger sequencing

After the identification of causative mutations in each case using the NGS platform (Table 1), all of the mutations identified using the NGS skeletal disorder panel were confirmed by Sanger sequencing and showed the expected familial segregation patterns when family members were analyzed.

Of the thirteen cases with identified variants, two (spondylocarpotarsal synostosis and Wolcott-Rallison syndrome) were autosomal recessive. Both were consanguineous cases, with homozygous mutations, which were present in the heterozygous state in both parents. The other 11 cases were either autosomal dominant or sporadic cases, including one *de novo* case of cleidocranial dysplasia. Mutation segregation was confirmed for six of these cases, parental samples were unavailable for the other four cases. On sequencing of the mutation regions in 105 samples from the normal Brazilian population, and in the 65 genetically independent cases, none of the mutations were detected. Also, the mutations were not found in the 1000 Genomes Project, nor in the Exome Variant Server or the ExAC browser, a database that spans more than 60.000 unrelated individuals sequenced as part of various disease-specific and population genetic studies [79].

## Discussion

The aim of the current work was to develop and apply a tool to be used in the molecular diagnosis of skeletal disorders, a group of heritable phenotypes characterized by a wide range of bone malformations and growth defects. The clinical diagnosis of these disorders is complicated by extensive phenotypic and genetic heterogeneity, with at least 450 disorders associated with more than 300 genes. Because of the heterogeneity and individual rarity of these disorders, we decided to simultaneously determine the sequences of all of the known genes associated with the skeletal disorders in each case. This allowed greater liberty in assembling samples for sequencing as it was not necessary to wait for cohorts of patients with the same or similar diagnostic hypotheses. In this study the NGS data were subsequently examined together with phenotypic features of each positive case in order to reach a definitive clinical and molecular diagnosis, validating the platform. Consequently, while future cases could benefit by integrating molecular data with clinical information, if such data are not available, it may be possible to rely on the molecular analysis alone. This hypothesis is supported by suspected skeletal disorder cases for which we succeeded in defining the diagnosis using sequence data.

Using the NGS panel it was possible to capture and sequence the exons of 309 genes and to analyze a group of 69 samples from patients with diverse skeletal phenotypes. The inter-run reproducibility together with the general consistency in data from different samples supports this multiplex method as an efficient and reliable first-tier diagnostic tool. The main drawback observed was the low to zero coverage of some targets, seen predominantly in first exons and high-GC regions. Similar data have been reported in other studies using hybridization capture [1, 80–82]. Future versions of the panel sequencing technology will require exon-specific approaches to fill in the missing exons. It is possible that reducing the inclusion of 5’-UTR sequences in future iterations may help reduce this problem.

The sequencing runs performed here showed 60–90% of reads on target, consistent with the proportion of sequences on target in custom capture kits in the literature that report 70–80% capture, depending on the size of the panel and the capture kit used [71, 72, 83]. The majority of off-target sequences reflected sequences adjacent to genes in the ROI and homologous genes. To increase the proportion of sequences on target, other capture and enrichment methods commercially available kits may be considered [83]. Mook *et*. *al* [73] used a second round of hybridization to increase the percentage of on-target capture. To prevent the loss of low complexity regions and thus missing mutations, studies have shown that it may help to decrease the concentration of clusters to 500–700 million/mm^2^ or avoid the PCR enrichment steps during the library preparation [84, 85].

Multiplexing 12 samples per run yielded between 100 and 140X of coverage, similar to the predicted theoretical coverage of 120X for this 1.44 Mb panel. The level of coverage obtained meant that, for many samples, more than 90% of targets could be analyzed with an average coverage greater than 20X. The coverage on this panel is similar to coverage reported in whole exome studies, while the cost per sample, as well as analysis time is greatly reduced [10, 86–93]. However, it should be noted that when the clinical diagnosis is well defined, even samples with lower average coverage can be successfully diagnosed by NGS. This was the case for one of our samples, in which a complete clinical definition allowed us to identify a pathogenic mutation in the *EIF2AK3* gene, with a depth of coverage of 34X (Table 1), despite such sample having only 34% of reads on-target with average coverage above 20X. In essence, when the clinical diagnosis makes it likely that the mutation will be in a gene included on the panel, it is more efficient in financial terms and timewise to use panel sequencing.

The main challenge that was faced in this work was related not to technical parameters but to the incomplete clinical characterization of patients. Several authors have pointed out that less than half of patients analyzed for genetic diseases receive a molecular definition of the disease [10, 94–99]. Our rate of molecular resolution for patients with a defined clinical diagnosis at the beginning was 22% (8/36), similar to the success rate for clinical genome and exome sequencing of 25% [8–10]. To improve this diagnosis yield, others have promoted algorithms for phenotype-driven bioinformatics analysis of patients [98]. For cases with a defined initial diagnostic hypothesis, but for whom no mutation was found in the expected gene, coverage was high. This was the case with cases diagnosed as multiple exostoses (exon coverage for *EXT1*, 60-215X; *EXT2*, 150X), geleophysic dysplasia (*ADAMTSL2*, 100-385X; *FBN1*, 115X), MOPDII (*PCNT* 95-690X), trichorhinophalangeal syndrome (*TRPS1*, 106-230X), Hajdu-Cheney syndrome (*NOTCH2*, 33-502X, including 201X for exon 34), and occulodentodigital syndrome (*GJA1*, 80X). Thus we can confidently rule out mutations in the listed genes for these patients, and suggest that the clinical diagnosis to be re-evaluated. In the case of the trichorhinophalangeal syndrome, gene deletions can cause the disease, which may not be detected by analysis of the panel. However, in our patient we found heterozygous SNPs in the gene, with close to 50% allele distribution, indicating that the gene is not deleted for this patient.

Many of the cases available to us were diagnosed based on clinical features alone, rather than on detailed analysis of x-rays, which is the gold standard for some skeletal disorders, mainly nonsyndromic diagnoses. For this reason, the filtering and prioritizing process resulted, in most cases, in several alterations that are predicted by the algorithms (PolyPhen, Mutation Taster) to be deleterious. This reflects the well-documented fact that the genome of any individual contains several alterations that are potentially disease causing [100, 101]. Because of uncertainties in the clinical diagnosis, we cannot be certain whether this means that the causative variant was filtered out or whether it was missed by the panel. To address this question will require obtaining radiographs on each case and working through the clinical diagnosis with each referring center.

The cases of suspected skeletal disorder (n = 20, S1 Table) were usually diagnosed simply as ‘osteodysplasia’, and the NGS panel identified the mutation in four cases (confirmed as spondyloepiphyseal dysplasia congenital type, hypochondroplasia, Apert syndrome and geleophysic dysplasia 2). This verifies that NGS can be used as a hypothesis-free approach for skeletal disorders [102]. For the rest of the cases in this group, candidate mutations may emerge from clinical and radiographic review in order to close the diagnosis.

For a further two cases, the molecular data were used to alter the initial diagnosis (one patient rediagnosed from Sotos syndrome to Weaver syndrome, another rediagnosed from Goldenhar syndrome to Townes-Brocks syndrome), an additional benefit of using the NGS approach, as the new diagnosis will provide better anticipatory counseling and potentially improved care.

These cases exemplify the goal of this project to assist the definitive diagnosis of skeletal disorders through the use of molecular and clinical data. With improved clinical characterization (especially analysis of x-rays in the case of skeletal disorders) the yield of definitive diagnoses could be much higher [2, 73, 103]. However, given the complexity of the skeletal disorders as a group and the difficulty of arriving at a clinical/radiographic diagnosis, the NGS approach has the potential to resolve cases even without extensive clinical information.

Of the 10 remaining cases diagnosed with syndromes for which there is as yet no known gene (S1 Table), no strong candidate mutations were identified, suggesting that at least some cases of these syndromes are caused by mutations in genes not yet associated with skeletal disorders. The NGS data consequently largely exclude the genes on the panel as being associated with these phenotypes.

As a deeper dataset is generated by sequencing additional cases using the panel, it is expected that variants that will be identified are not the primary cause of the phenotype. These include possible SNPs that have not been previously described, heterozygosity for recessive mutations and potential hypomorphic alleles. The occurrence of phenotypic variability in skeletal disorders, including between patients with the same phenotype caused by mutations in the same gene, is well known [79, 104–106]. These secondary sequence alterations are candidates for modifier loci that may alter phenotypic expressivity, however the confirmation of their impact will be complex, especially owing to the rarity of these phenotypes. While modifier loci could theoretically exist in any part of the genome, it is not unreasonable to suggest that alterations in genes important to skeletal development may be prime candidates [2]. Furthermore, analysis of datasets such as this one will allow exploration of the possibility of a digenic basis for some recessive cases [107].

There are almost 100 skeletal disorders of unknown etiology [23], but the pace at which the molecular basis of these phenotypes are being identified is rapid. Indeed, since the development of this gene panel several new causative genes for skeletal disorders have been characterized, so the panel will need to be continuously updated to keep pace with new developments. Still, for the clinical diagnosis of the most common skeletal disorders, or for molecular screening studies of more complex diseases, the panel becomes feasible, since it contains the majority of the causative genes of known skeletal disorders. This approach has limitations similar to those encountered in other NGS approaches. It is not very suitable for detection of duplications and deletions, especially in extra-genic regions, such as the *SOX9* control regions that may cause campomelic dysplasia when deleted. In addition, any low-coverage regions must be detected and re-sequenced for complete confidence in the sequencing data.

In common with several other published studies, panel sequencing was found to be a rapid and cost-efficient solution for genetically heterogeneous skeletal conditions [2, 17, 80]. Our approach proved to be effective for the molecular analysis of skeletal disorders, with the goal of sequencing all exons of all genes known to be associated with these disorders. We believe this is an advantage over the skeletal disorders panels offered by commercial laboratories, which are more limited and higher cost. Although our approach involved sequencing of many genes not relevant to a particular case, the benefit of doing so is that many cases with distinct phenotypes can be analyzed simultaneously, allowing more flexibility of patient inclusion. The method described is faster, more reliable and cheaper than the Sanger method (per gene/case) and avoids the need for several sub-panels of genes. A strong multidisciplinary collaboration with medical professionals during the selection of samples might improve performance, but even without such information diagnostic molecular data can be obtained.

## Supporting Information

S1 Fig Pattern of coverage distribution in sequenced targets. Most common distribution pattern: the target extremities have a lower coverage than the center. Samples varied principally in level of average coverage, which has the effect of shifting the curve vertically, with little variation in curve shape.
(EPS)

S1 Table List of patients and their respective clinical data and medical diagnoses. (XLS)

S2 Table List of the 309 selected genes known to be involved in genetic disorders of the skeleton, used in the gene panel design, alongside the associated phenotypes. (XLS)
